# Root Skewing-Associated Genes Impact the Spaceflight Response of *Arabidopsis thaliana*

**DOI:** 10.3389/fpls.2020.00239

**Published:** 2020-03-04

**Authors:** Brandon Califar, Natasha J. Sng, Agata Zupanska, Anna-Lisa Paul, Robert J. Ferl

**Affiliations:** ^1^Horticultural Sciences, University of Florida, Gainesville, FL, United States; ^2^The Genetics Institute, University of Florida, Gainesville, FL, United States; ^3^Program in Genetics and Genomics, University of Florida, Gainesville, FL, United States; ^4^Program in Plant Molecular and Cellular Biology, University of Florida, Gainesville, FL, United States; ^5^Interdisciplinary Center for Biotechnology and Research, University of Florida, Gainesville, FL, United States

**Keywords:** spaceflight, root, skewing, transcriptomics, abiotic stress, acclimation, physiological adaptation

## Abstract

The observation that plant roots skew in microgravity recently refuted the long-held conviction that skewing was a gravity-dependent phenomenon. Further, spaceflight root skewing suggests that specific root morphologies and cell wall remodeling systems may be important aspects of spaceflight physiological adaptation. However, connections between skewing, cell wall modification and spaceflight physiology are currently based on inferences rather than direct tests. Therefore, the Advanced Plant Experiments-03-2 (APEX-03-2) spaceflight study was designed to elucidate the contribution of two skewing- and cell wall-associated genes in Arabidopsis to root behavior and gene expression patterns in spaceflight, to assess whether interruptions of different skewing pathways affect the overall spaceflight-associated process. SPIRAL1 is a skewing-related protein implicated in directional cell expansion, and functions by regulating cortical microtubule dynamics. SKU5 is skewing-related glycosylphosphatidylinositol-anchored protein of the plasma membrane and cell wall implicated in stress response signaling. These two genes function in different cellular pathways that affect skewing on the Earth, and enable a test of the relevance of skewing pathways to spaceflight physiological adaptation. In this study, both *sku5* and *spr1* mutants showed different skewing behavior and markedly different patterns of gene expression in the spaceflight environment. The *spr1* mutant showed fewer differentially expressed genes than its Col-0 wild-type, whereas *sku5* showed considerably more than its WS wild-type. Developmental age played a substantial role in spaceflight acclimation in all genotypes, but particularly in *sku5* plants, where spaceflight 4d seedlings had almost 10-times as many highly differentially expressed genes as the 8d seedlings. These differences demonstrated that the two skewing pathways represented by *SKU5* and *SPR1* have unique and opposite contributions to physiological adaptation to spaceflight. The *spr1* response is less intense than wild type, suggesting that the loss of SPR1 positively impacts spaceflight adaptation. Conversely, the intensity of the *sku5* responses suggests that the loss of SKU5 initiates a much more complex, deeper and more stress related response to spaceflight. This suggests that proper SKU5 function is important to spaceflight adaptation.

## Introduction

Spaceflight studies offer unique insights into plant biological processes, and enable the exploration of the relationships between root morphology, gene expression and the physiological adaptation to spaceflight. The developmental patterns of plant organs are continuously altered through perception of the environment, signal integration, and response to environmental stimuli. A diversity of tropic gradients influence the path of growth in roots by initiating localized, asymmetrical changes in cell elongation. These changes are primarily brought about through hormonal interactions and subsequent remodeling of cell physiology (Roy and Bassham, 2014; Vandenbrink et al., 2014; Schultz et al., 2017). Root skewing and waving are phenomena in which root growth deviates from a gravity vector throughout its development, and which vary between the *Arabidopsis thaliana* (Arabidopsis) ecotypes Columbia (Col-0) and Wassilewskija (WS) (Rutherford and Masson, 1996; Roy and Bassham, 2014; Schultz et al., 2017). Although once thought to be a gravity-dependent growth behavior (Oliva and Dunand, 2007), skewing occurs in the microgravity of spaceflight (Paul et al., 2012a). This suggests that skewing is independent of both the tropic force of gravity and the gravity-induced contact forces between roots and growth media (Millar et al., 2011; Paul et al., 2012a, 2013; Nakashima et al., 2014). Therefore, the spaceflight environment provides a unique and relevant context in which to study genes associated with skewing phenotypes.

Plants grown in the spaceflight environment exhibit complex and unique gene expression patterns (e.g., Paul et al., 2005; Paul et al., 2012b, 2013, 2017; Correll et al., 2013; Kwon et al., 2015; Johnson et al., 2017; Choi et al., 2019). The predominant feature of the spaceflight environment is microgravity. The lack of gravity has a direct effect on plant cells and signal transduction; this direct effect represents a novel environment for plants, and any terrestrial organism, and appears to be generally perceived as stressful. In addition, microgravity imposes secondary environmental stresses due to the disruption of fluid movement, and any processes influenced by convection, such as gas exchange and temperature redistribution. Combinatorial stresses induce changes in gene expression and tolerance that are not fully recapitulated by exposure to the individual stressors (e.g., Ramegowda and Senthil-Kumar, 2015; Suzuki et al., 2016). Thus it is important to think of the spaceflight environment as more complex than microgravity alone. Several classes of stress response genes have been identified as consistently involved in the response to spaceflight across ecotypes of Arabidopsis via analyses of gene expression. Heat shock genes are often induced by spaceflight (Paul et al., 2005, 2012b; Salmi and Roux, 2008; Shagimardanova et al., 2010; Zupanska et al., 2013, 2017, 2019; Johnson et al., 2017; Choi et al., 2019). Reactive oxygen species (ROS) signaling and scavenging processes are also common in the spaceflight response, though ROS-associated genes have been observed as both up- and downregulated in spaceflight (Shagimardanova et al., 2010; Correll et al., 2013; Paul et al., 2013, 2017; Kwon et al., 2015; Choi et al., 2019; Zhou et al., 2019). Some stress gene expression changes are also associated with spaceflight-induced changes in the Arabidopsis methylome (Zhou et al., 2019). Cell wall remodeling processes are enriched in spaceflight gene expression datasets (Paul et al., 2012b, 2013, 2017; Correll et al., 2013; Kwon et al., 2015; Johnson et al., 2017). Cell wall remodeling genes, typically associated with biotic stress and pathogen defense pathways, also contribute to spaceflight acclimation (Paul et al., 2012b, 2013, 2017; Correll et al., 2013; Choi et al., 2019). Spaceflight also affects abundances of proteins of defense pathways and cell wall remodeling (Mazars et al., 2014; Ferl et al., 2015). However, the particular genetic pathways activated and repressed in spaceflight vary among ecotypes of Arabidopsis, demonstrating that there are significant genotypic contributions to spaceflight physiological adaptation (Paul et al., 2017; Beisel et al., 2019; Choi et al., 2019).

Mutations in genes associated with stress response and signaling pathways can significantly alter the differential gene expression profiles of spaceflight physiological adaptation. Single gene mutations in heat shock transcription factors, gravity perception genes, and light signaling genes in Arabidopsis seedlings and cultures exhibit altered spaceflight responses (Paul et al., 2017; Zupanska et al., 2017, 2019). Therefore, we sought to understand better the relationships among spaceflight responses, root morphology, and spaceflight adaptation by exploring the spaceflight responses of two single gene skewing-related mutant lines: *spiral1* and *sku5*.

*Spiral1* (*Spr1*) is a skewing-related gene that contributes to the process of anisotropic cell expansion by regulating cortical microtubule dynamics. The *spr1* mutation results in axial rotation of cell files throughout the plant, which manifests in the roots as skewing to the left when viewed, according to convention, from beneath their growth medium (Rutherford and Masson, 1996; Furutani et al., 2000; Nakajima et al., 2004; Sedbrook et al., 2004; Galva et al., 2014). The SPR1 protein is a microtubule plus-end tracking protein that localizes to the plus ends of actively polymerizing cortical microtubules, dissociating upon a shift to microtubule depolymerization (Sedbrook et al., 2004; Galva et al., 2014). This association is mediated by two similar motifs on the N- and C-termini of the protein, which allow SPR1 to act as an intermolecular linker (Nakajima et al., 2004; Sedbrook et al., 2004; Galva et al., 2014). The protein END-BINDING 1B (EB1b) co-localizes with SPR1 at the microtubule plus-end, where each protein interacts with the other as well as tubulin subunits (Galva et al., 2014). This creates the dual effects of SPR1 and EB1b increasing microtubule stability and polymerization rate, while also enhancing the rescue rate of depolymerizing microtubules (Galva et al., 2014). However, the *spr1* mutation also leads to a decreased rate of shrinkage among depolymerizing microtubules (Galva et al., 2014). The regulation of microtubule dynamics is critical to many environmental responses, such as in salt stress where 26S proteasome-mediated degradation of SPR1 is known to occur in conjunction with microtubule reorganization to enable stress acclimation (Shoji et al., 2006; Wang et al., 2011; Chen et al., 2016). In addition to these micro-scale changes, the skewing phenotype of *spr1* is significantly enhanced by cold treatment, and suppressed by both salt and heat stresses (Furutani et al., 2000; Sedbrook et al., 2004). This connects *SPR1* to processes regulating morphology in both optimal and stressful environments.

*Sku5* is a skewing-associated gene that encodes a protein in the SKU5-SIMILAR (SKS) family, which is related to known Arabidopsis copper oxidases, but which contains only one Type-II copper-coordinating domain of unknown specificity (Sedbrook et al., 2002). The *sku5* mutant was originally noted for the rightward skewing it exhibits on vertically oriented growth media, which manifests in both WS and Col-0 backgrounds (Sedbrook et al., 2002; Swarbreck et al., 2019). *Sku5* seedlings exhibit reductions of root length but not of cell size, and SKU5 is hypothesized to act in the process of cell division as a result (Sedbrook et al., 2002). SKU5 localizes to the plasma membrane, but is also present in soluble and cell wall-binding forms in the extracellular milieu (Sedbrook et al., 2002; Borderies et al., 2003; Baral et al., 2015; Chen et al., 2018). SKU5 is a glycosylphosphatidylinositol-anchored protein (GPI-AP), and as such is a component of discrete plasma membrane nanodomains known as “lipid rafts” (Borner et al., 2005; Elortza et al., 2006; Chen et al., 2018; Mamode Cassim et al., 2019). These nanodomains of the plasma membrane and their associated GPI-APs play roles in stress response signaling at the interface between the cell wall and membrane, where they are envisioned to help shape the “signatures” that activate downstream plant adaptive responses to the stresses they experience (Minami et al., 2008; Takahashi et al., 2013, 2016, 2019; Shabala et al., 2015; Yeats et al., 2018; Miki et al., 2019). The endocytosis of SKU5 and other GPI-APs is modulated across the root tip by salt and auxin treatments, linking the movement of these proteins to stress responses (Baral et al., 2015). The expression of SKU5 is itself regulated by boron deprivation, cold, salt, and immune responses, as well as abscisic acid (ABA) treatment (Keinath et al., 2010; Elmore et al., 2012; Li et al., 2012; Tanaka et al., 2016; Shi et al., 2018; Miki et al., 2019). While SKU5 interactors are unknown in Arabidopsis, a maize homolog of SKU5 interacts with a C-terminal peptide of AUXIN-BINDING PROTEIN 1 (ABP1) *in vitro* (Shimomura, 2006). ABP1 is associated with auxin signaling pathways that activate the plant TARGET OF RAPAMYCIN (TOR) regulatory complex, remodel the cortical microtubule network, and induce expansive growth by triggering cation influx (Xu et al., 2014; Chen et al., 2016; Dahlke et al., 2017; Schepetilnikov et al., 2017). This further connects SKU5 to characterized pathways which affect stress responses and regulate the balance between growth and autophagic processes under stressful conditions, such as that of spaceflight.

SKU5 and SPR1 are therefore very different proteins in seemingly unrelated physiological processes, but both proteins have roles in root skewing. Both of these proteins are also associated with cell wall remodeling, a process that is regularly involved in spaceflight acclimation. As such, these genes offer an appropriate initial genetic dissection of root skewing and the effect of skewing pathways in the microgravity of spaceflight environments. Extensive discussion of the relevance of skewing and waving in microgravity has been presented elsewhere (e.g., Paul et al., 2012a), but to paraphrase from that paper, prior to spaceflight research, the consensus was that gravitropism was the directional driver in skewing (e.g., Simmons et al., 1995; Oliva and Dunand, 2007). Yet we now know that gravity is not required, and root skewing in spaceflight appears to be an inherent feature of many Arabidopsis ecotypes, even in conditions lacking both light and gravity (Millar et al., 2011; Paul et al., 2012a, 2017; Nakashima et al., 2014). These observations suggest a testable relationship among root morphologies governed by skewing regulation pathways. The hypothesis is that skewing pathways play a large role in spaceflight adaptation via the inherent cell wall remodeling that accompanies those morphologies and the physiological adaptation to spaceflight. This relationship was examined by growing *spr1*, *sku5* and their wild type controls for 4 and 8 days on the International Space Station and at the Kennedy Space Center, then observing both their growth morphologies and gene expression profiles in response to spaceflight as measures of response quality and complexity. A change in gene expression or morphology during spaceflight would suggest a significant role for these skewing genes in the physiological adaptation process.

Differential gene expression profiles are often deployed as measures of the underlying gene expression changes needed for physiological adaptation and developmental changes within an organism. Differential gene expression responses, in terms of the number of genes involved and their fold-change levels, may be considered a measure of the metabolic cost of adapting to that environment (e.g.: Chan et al., 2016). Therefore gene expression profiles morphologies were used to examine the relationships between skewing genes and the resulting complexity of spaceflight acclimation. SKU5 and SPR1 function in different cellular pathways that affect skewing on the Earth, which enabled a test of the relevance of contrasting skewing pathways to spaceflight physiological adaptation. Growth morphologies were used to examine the productivity and developmental success of these genotypes in spaceflight.

## Materials and Methods

### Plant Material and Plate Setup

*Arabidopsis thaliana* wild ecotype Columbia (Col-0, CS70000) seed stock, and T-DNA insertion lines for the *spr1* (CS6547 – Col-0 background) and *sku5* (CS16268 – WS background), were acquired from the Arabidopsis Biological Resource Center (ABRC) (arabidopsis.org; Lamesch et al., 2011). The wild type Wassilewskija (WS) line used in this study was propagated in our laboratory for more than 25 years. This WS line has been used in multiple spaceflight studies (Paul et al., 2012a, 2013, 2017; Zhou et al., 2019), and seed samples are available upon request. The ABRC denotes WS as stock CS915. Petri dishes (100 mm × 15 mm; Fisher Scientific, Pittsburgh, PA, United States), containing 50 mL of a 0.5% Phytagel-based growth medium supplemented with: 0.5× Murashige-Skoog salts, 0.5% (w/v) sucrose, and 1× Gamborg’s Vitamin Mixture, were prepared aseptically for planting. Seeds were sterilized and planted according to methods allowing for the maintenance of seed dormancy (Sng et al., 2014). Briefly, seeds were stored with Desiccant-Anhydrous Indicating Drierite (W.A. Hammond Drierite Company, stock #24001) for 1 week before sterilization. The seeds were sterilized with 70% ethanol for 10 min and dried in a laminar flow hood. Seeds were stored at 4°C in sterile screw-cap microcentrifuge tubes until planting. Seeds were suspended in sterile water and dispensed onto the media surface. Approximately 12–15 seeds were planted in a row on each plate, with one row on the 8-day (8d) plates and two rows on the 4-day (4d) plates. At time of harvest, each genotype and age was represented by 9–25 viable, replicate seedlings. All replicates contributed to morphological observations, whereas the transcriptome analyses were conducted with four biological replicates, comprising 5–8 4d plant roots, or 2–3 8d plant roots. The plates were sealed with Micropore^®^ (3M, Maplewood, MN, United States) tape, and wrapped in Duvetyne Black-Out Fabric (Seattle Fabrics). The time from suspending dry seeds in water to the completed wrapping of the plate in Duvetyne was less than 10 min; this timing was essential to maintain seed dormancy. Wrapped plates were then transported to the Kennedy Space Center (KSC, FL, United States) under cold stowage, maintaining temperatures of 4–10°C until launch.

### Spaceflight Experimental Workflow

**FIGURE 1:**
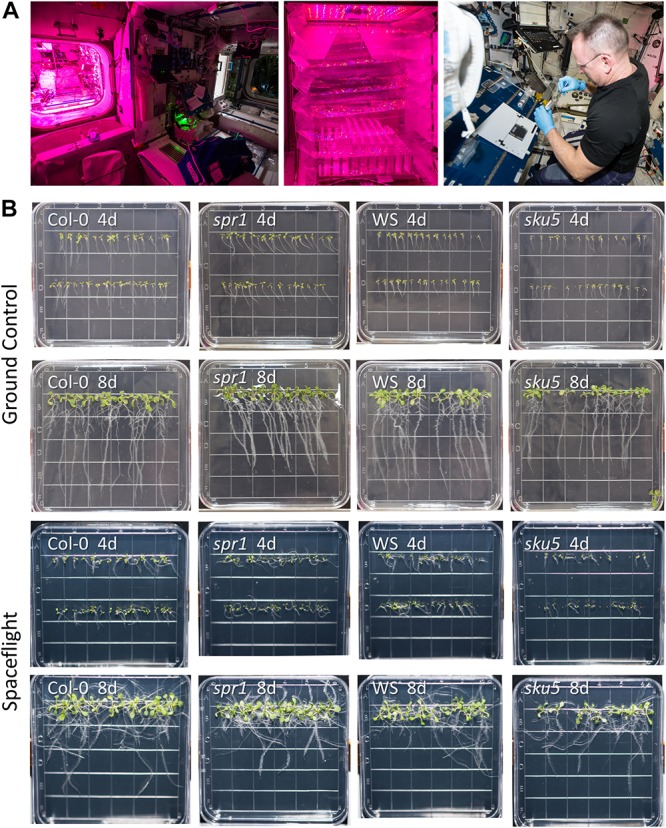
Advanced Plant Experiments-03-2 overview. **(A)** Images detailing the standard procedures used onboard the ISS during the experiment. In Panel 1, the Vegetable Production System (VPS, “Veggie”) and its local environment can be seen, while panel 2 provides a close-up of the plate setup inside Veggie after insertion. Panel 3 shows astronaut Butch Wilmore harvesting seedlings into a KFT (Kennedy Space Center Fixation Tube), and the workstation used for the orbital harvests. The images of the Veggie hardware and Butch Wilmore were taken on the ISS by NASA; all NASA photos are in the public domain. **(B)** Full images of plates taken at the time of harvest and from which tissue was used in the APEX-03-2 RNA-Seq experiment. Images from the ground control are in the top two rows, while those from the spaceflight experiment are in the bottom two rows. The genotype and developmental age of the seedlings are listed on each image. The provided images were taken from above the surface of the growth medium.

The Advanced Plant Experiments 03-2 (APEX-03-2) study, also known under NASA Operational Nomenclature as the Transgenic Arabidopsis Gene Expression System – Intracellular Signaling Architecture (TAGES-ISA) study, has been previously described (Ferl and Paul, 2016; Beisel et al., 2019; Zhou et al., 2019). The SpaceX CRS-5 mission, carrying the dormant, seeded growth plates as part of its cargo, was launched to the International Space Center (ISS) from KSC on January 10, 2015. Capsule docking and transfer of materials to the ISS occurred on January 12 and 13, respectively. The growth plates were removed from cold stowage onboard the ISS on January 26, unwrapped, and inserted into the Vegetable Production System (VPS, colloquially “Veggie”) perpendicular to the light bank (Figure 1A). During the growth period, constant lighting at a level of 100–135 μmol m^–2^ s^–1^ was used. Each age and genotype was represented by a 10 cm Petri plate. There were 12–15 seedlings on the 8-day-old plates (8d) and 35–40 seedlings on the 4-day-old plates (4d). The plates were removed from the Veggie plant growth hardware after 4 and 8 days of growth, at which time they were photographed by the crew. The seedlings of each plate were then harvested into individual KSC Fixation Tubes (KFTs) pre-loaded with RNAlater^TM^ (Ambion, Grand Island, NY, United States) preservative (one plate per KFT) (Figure 1A). Actuation of the KFT submerged the seedlings in RNAlater and sealed the tube. The harvest tubes remained at ambient temperature for 12 h to allow full perfusion of the tissues, and were then transferred to the MELFI −80°C freezer. The samples were transferred from the MELFI to cold stowage onboard the Dragon capsule on February 9^th^ and remained frozen in transit to KSC for de-integration on February 15, at which time the samples were transferred to 50 mL Falcon tubes with minimal thawing. The samples were transferred to the Principal Investigators and were transported to the University of Florida, where they were kept under −80°C storage.

The ground control (GC) for the experiment, composed of an identical set of plants and plates, was performed on a 48-h delay at KSC using Veggie hardware within the ISS Environmental Simulator (ISSES) chamber. The same growth timeline was used, with seedlings being imaged and harvested into RNAlater-containing KFTs at 4d and 8d time points. The GC operations were as described above for the spaceflight experiment, with KSC personnel following the precise timing of the astronaut activities. Telemetry data also enabled the ISSES chamber to replicate the CO_2_ levels, temperature, and ambient lighting in the vicinity of the Veggie hardware onboard the ISS across the course of the experiment.

The consistency of operations between spaceflight and GCs was also checked against video data captured on the ISS. Over-the-shoulder videos of astronaut activities for the 4d and 8d harvests were examined for timing of operations and showed that spaceflight seedling harvests were not disproportionately treated during the process at either time point or compared to GC harvests. The plates were harvested in comparable windows of time, ensuring that no additional stress, such as drought stress, was introduced into the harvest process (Supplementary Table S1).

### RNA Isolation

The spaceflight and GC seedlings, stored in RNAlater, were transferred from −80°C storage to 4°C overnight to thaw. Seedlings in RNAlater were examined with an Olympus SZX12 stereoscope (Olympus Corporation, Tokyo, Japan) and whole roots were dissected away from the shoot and hypocotyl. The remaining shoot and hypocotyl tissues were restored to −80°C in RNAlater. RNA was prepared from 5–8 4d plant roots, and 2–3 8d plant roots. Total RNA was extracted using the Qiashredder and RNAeasy kits from QIAGEN (QIAGEN Sciences, MD, United States) according to the manufacturer’s instructions. An on-column digestion with RNase-free DNase (QIAGEN GmbH, Hilden, Germany) was used to remove residual DNA.

### Library Preparation

Library preparation was performed at the University of Florida’s Interdisciplinary Center for Biotechnology Research (ICBR) Gene Expression Core. RNA integrity was verified using the Agilent 2100 BioAnalyzer (Agilent Technologies, Santa Clara, CA, United States). Of the total RNA, 10 ng were used to construct cDNA libraries with the ClonTech SMART-Seq v4 ultra-low input RNA kit for sequencing (Clontech Laboratories, Inc., Cat#: 634890), according to the manufacturer’s instructions. Briefly, 1st strand cDNA was primed by the SMART-Seq v4 oligonucleotide, which then base-pairs with these additional nucleotides, creating an extended template. The reverse transcriptase then switches templates and continues transcribing to the end of the oligonucleotide, resulting full-length cDNA that contains an anchor sequence that serves as a universal priming site for second strand synthesis. Then, cDNA was amplified with primer II A for 8 PCR cycles. Illumina sequencing libraries were then generated with 150 pg of cDNA using the Illumina Nextera DNA Sample Preparation Kit (Cat#: FC-131-1024) according to the manufacturer’s instructions. Briefly, 150 pg of cDNA were fragmented by tagmentation reaction and adapter sequences were added onto template cDNA by PCR amplification. Libraries were quantitated using both the 2100 BioAnalyzer and qPCR (Kapa Biosystems, catalog number: KK4824).

### RNA-Seq

Sequencing experiments were performed at the UF ICBR Next-Generation DNA Sequencing Core. In preparation for sequencing, barcoded libraries were sized on the 2100 BioAnalyzer, and quantitated by QUBIT (Thermo Fisher Scientific, Waltham, MA, United States) and qPCR (Kapa Biosystems, catalog number: KK4824). Individual libraries were pooled equimolarly at 4 nM. This “working pool” was used as input in the NextSeq500 instrument sample preparation protocol (Illumina, Part#: 15048776, Rev A). Typically, a 1.3 pM library concentration resulted in an optimal clustering density in our instrument (i.e., ∼200,000 clusters per mm^2^). Samples were sequenced on 5 flow cells (5 NextSeq500 runs), using a 2 × 75 cycles (paired-end) configuration. A typical sequencing run in the NextSeq500 produced 750–800 million paired-end reads with a Q30 ≥ 85%. For RNA-Seq, around 40 million paired-end reads per sample provided sufficient depth for transcriptome analysis (Tarazona et al., 2011).

### Bioinformatic Analysis

Bioinformatic processing of the sequencing data was performed at the UF ICBR Bioinformatics Core. Fastq files were trimmed to remove sequencing adapters and low-quality base calls using Trimmomatic (version 0.36) with the parameters: LEADING:3, TRAILING:3, SLIDINGWINDOW:4:15, MINLEN:50 (Bolger et al., 2014). Quality control of the trimmed reads was performed using FastQC (version 0.11.2) (Andrews, 2010). Reads for each set of Arabidopsis lines were aligned to their respective reference genomes using the STAR aligner (version 2.5.1b) (Dobin et al., 2012). The Col-0 TAIR10 genome release was used for reads from Col-0 and *spr1* (Lamesch et al., 2011; Berardini et al., 2015), while reads from WS and *sku5* were aligned to the WS reference genome (Gan et al., 2011). Duplicate reads resulting from PCR artifacts were then removed using the Picard MarkDuplicates tool (Broad Institute, 2019b). In total, 502 million transcriptomic reads were aligned. Following alignment, expression quantification and differential gene expression analysis were performed using Cufflinks and Cuffdiff (version 2.2.1), respectively, with default parameters (Trapnell et al., 2010, 2012). Output from Cuffdiff was then parsed with custom scripts to generate the final annotated tables of differentially expressed genes (DEGs). A cut-off false-discovery rate (FDR) of 0.05 was used for calling statistical significance in differential gene expression

### RNA-Seq Data Analysis

Tables of DEGs for each comparison were combined and analyzed through Microsoft Excel, in order to generate lists of Arabidopsis Genome Initiative (AGI) identifiers with their corresponding [Log_2_(Fold Change)] values. Machine annotation of AGI ID lists was carried out using G:Profiler (Raudvere et al., 2019), while the TAIR (Lamesch et al., 2011; Berardini et al., 2015) and Araport ThaleMine (Krishnakumar et al., 2016) databases were used to investigate genes of interest. DEGs of a comparison (e.g., FLT vs. GC) with a greater than twofold change in at least one context of the comparison were retained for analysis (Supplementary File S1). This was done in order to minimize the effect instituting a cut-off would have on analyzing the expression patterns of a gene. Significant DEGs with a fold-change of “inf” or “-inf” were retained and assigned a [Log_2_(Fold Change)] value of 10 for heat-mapping, as they have a consistent FPKM of zero in one context but are consistently detected at a significantly higher level in the opposing context. Gene ontology (GO) analyses of only those DEGs with a greater than twofold change were carried out using the lists of their AGI IDs, separated by their up- or downregulation, with the PANTHER statistical overrepresentation tool (Mi et al., 2018). GO terms for each comparison were separated into those unique to one context and those overlapping between contexts, and the output is available in Supplementary File S2. Of those resulting, all GO terms overlapping between contexts were retained for heatmapping. Either one-half or five, whichever was greater, of each context’s unique GO terms were retained after sorting by *q*-value from most to least significant. GO term lists were then manually pruned in order to reduce highly redundant information (e.g., “cellular response to chemical” and “response to chemical”) only when the redundant terms demonstrated the same pattern of over- or underrepresentation across contexts. Complete lists of genes annotated to specific GO Terms of interest were gathered from AmiGO2 (Carbon et al., 2008). Heatmaps were then generated using DEG [Log_2_(Fold Change)] values or GO term [-Log(*q*-value)] values as input for the Morpheus webtool (Broad Institute, 2019a).

### Measurements of Seedling Roots

Germination rates and the growth of roots in each environment were derived from images taken of the growth plates at the times of harvest (Figure 1B). Germination rates were assessed by counting the number of seedlings and ungerminated seeds from the 4-day images taken just prior to harvest. Statistical significance of germination rate changes were assessed using χ^2^ tests with the conservative Yates correction (Yates, 1934). The comparisons made were between mutant and wild-type lines, and between spaceflight and GC growth conditions. To measure roots, images were analyzed using the FIJI distribution of ImageJ (Schindelin et al., 2012; Rueden et al., 2017). The JFilament plugin was used to track primary roots, creating a set of points describing each root (Smith et al., 2010). These data were processed using an R script that provided the length of each primary root as output, alongside other measures (Schultz et al., 2016; R Core Team, 2019). Lengths were corrected for scale based on the pixel length of the Petri dish grid-squares to allow for comparisons between images. Differences in root length were assessed through two-tailed Student’s *t*-tests, with the Bonferroni correction for multiple testing applied (Snedecor and Cochran, 1989). The comparisons made were between spaceflight and GC seedlings for each genotype, and between each skewing mutant and its respective wild-type. As conventional measurements of angles of root growth are dependent upon a reference gravity vector, the roots of 4-day-old seedlings were measured manually using FIJI (Schindelin et al., 2012; Rueden et al., 2017). The angle between the root tip and the growth direction of the beginning of the root was used. Positive and negative angles were used to represent rightward and leftward changes in directionality, respectively, relative to the initial direction of growth when viewed from below the growth media. Angles were plotted using the ggplot2 package in R (Wickham, 2016). In these polar plots, the magnitude of a bar corresponds to the number of roots within a particular bin, with the placement of the bar corresponding to the measured angle. Differing upper limits were used for spaceflight (0–20) and GC (0–10) plots in order to allow the many lower-magnitude bins in the spaceflight plots to be visualized by effectively zooming-in on the data. However, the scaling between inner rings is consistent between plots. As such, a bar meeting the second ring at the −25° line indicates that ten roots had an angle shift between 15° and 30° to the left during their growth.

## Results

**FIGURE 2:**
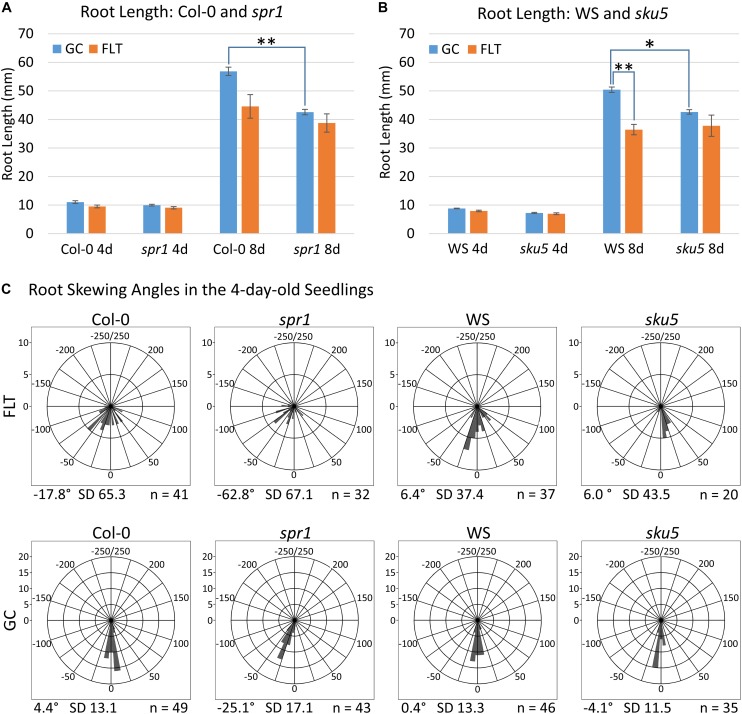
Quantification of APEX-03-2 Root Growth. Images acquired at the time of harvest of spaceflight (FLT) and ground control (GC) plants were used for measurements. Primary root length measurements for **(A)** Col-0 and *spr1* and **(B)** WS and *sku5* were taken using Jfilament and analyzed in R. Significant differences between genotypes (*spr1* vs. Col-0, *sku5* vs. WS) and growth conditions (FLT vs. GC) were assessed via two-tailed *t*-tests and are indicated by asterisks above bars connecting the relevant sets of measurements (Bonferroni correction: ^∗^*p* < 0.0125; ^∗∗^*p* < 0.001). The 8d Col-0 FLT vs. GC comparison did not meet this cutoff (*p* = 0.0151). Error bars show the SEM of each set of measurements. **(C)** The angle measured between the root tip and the initial direction of root growth in the 4-day-old seedlings. Plots are separated into rows by growth condition, and into columns by genotype. Positive and negative angles represent rightward and leftward growth of roots, respectively, when viewed from behind the media. Angles are binned in 10° intervals, with each bar’s length representing the number of roots within that bin. As such, a bar reaching the second ring of each plot represents ten roots within that bin. However, note that the upper limit of bin size is lowered in the spaceflight plots to accommodate the higher variation in these data. The total number of roots represented in each plot is indicated in its bottom-right corner, and the mean angle of each plot is noted in its bottom-left corner. FLT, spaceflight; GC, ground control.

APEX-03 seedling growth in occurred in the Veggie hardware on the ISS (Figure 1A). Veggie is housed in the Columbus module (Figure 1A, left), and for the APEX-03 experiment, configured to accommodate racks of 10 cm Petri plates (Figure 1A, middle). Images of plant growth and morphology were recorded by astronaut Butch Wilmore just prior to harvest (Figures 1A, right, B). Col-0 and WS can display distinct patterns of root growth in response to environmental stimuli. In terrestrial environments, changing the angle of the growth surface can create ecotype-specific patterns of skewing (Rutherford and Masson, 1996; Schultz et al., 2017). In a microgravity environment with a gradient light source to impart a tropic cue, skewing patterns of WS and Col-0 recapitulate the patterns seen on terrestrial angled growth surfaces (Paul et al., 2012a). However, in a microgravity environment with uniform, non-directional lighting such as provided by the Veggie hardware in the current experiment, root growth patterns are more disorganized (Figure 1B). Nonetheless, skewing trends can still be discerned by following the angles of growth as the roots develop, and these are quantified in Figure 2A for Col-0 and *spr1*, and in Figure 2B for WS and *sku5*. In all representations and discussions of skewing, the direction indicated is from the perspective of being viewed from behind the growth medium.

### Col-0 and *Spr1*

#### Germination and Morphology of *spr1* in Spaceflight

**FIGURE 3:**
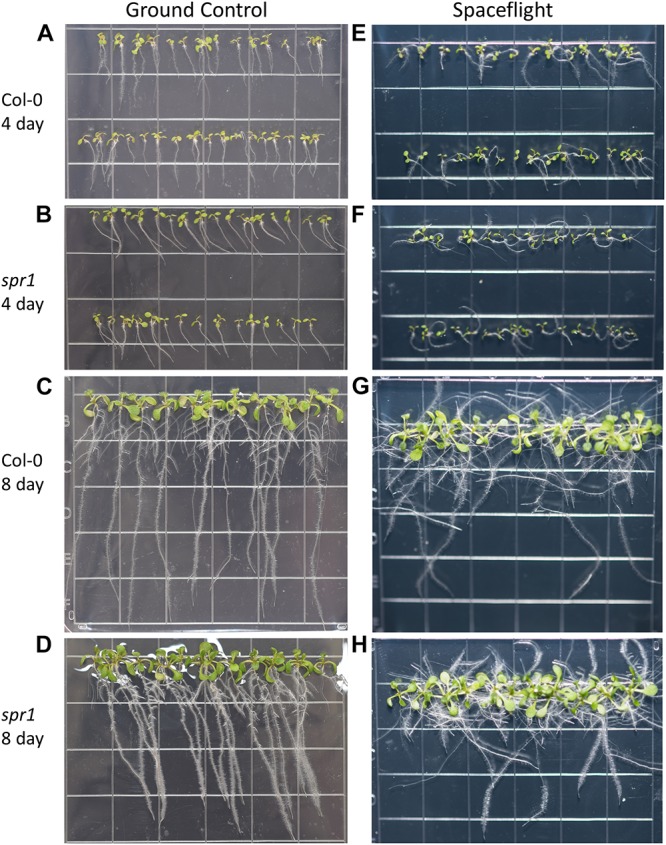
High resolution images of the Col-0 and *spr1* growth patterns. Images are arranged such that the ground control seedlings **(A–D)** are in the left column while spaceflight seedlings are on the right **(E–H)**. Vertically, images alternate between Col-0 **(A,C,E,G)** and *spr1* **(B,D,F,H)**. The images taken at the 4 day time point **(A,B,E,F)** are grouped above those taken at the 8 day time point **(C,D,G,H)**. Images taken from above the growth medium at the time of harvest. Grid squares are 13 mm wide.

Phenotypic differences among the Col-0 background plants in GC and spaceflight (FLT) environments were primarily limited to skewing angles in the roots (Figures 2, 3). There were no statistically significant differences in the primary root length between GC and FLT in either Col-0 or *spr1* 4d or 8d plants. Although Col-0 root length appeared to be reduced in spaceflight in 8d plants, the *p-*value did not quite meet the requirements for significance (two-tailed *t*-test, *p* = 0.0152, whereas cut-off was *p* < 0.0150) (Figure 2A). There were differences in root length between Col-0 and *spr1* genotypes in the GC (*p* = 1.48 E-9) but FLT Col-0 and FLT *spr1* roots were not statistically different.

Both 4d and 8d GC Col-0 seedlings grew with very little left or right deviation down the vertical face of the plate medium, while the GC *spr1* mutant plants of both ages showed distinct skewing to the left (Figures 2C, 3A–D). The 4d FLT Col-0 plants generally skewed slightly to the left with some variation (Figures 2C, 3E). In contrast, the roots of the 4d FLT *spr1* plants demonstrated an increased severity of skewing, which was manifested as a strong left-hand curved growth pattern (Figures 2C, 3E,F) that was significantly different between GC and FLT (two-tailed *t*-test, *p* = 0.00385). For both genotypes the variance in the growth angle (as noted in the SD values in Figure 2) was greater in the spaceflight samples. After 8d of growth, the increased skewing in FLT *spr1* led to associations with other roots, in contrast to FLT Col-0 which tended to spread more evenly across the plate (Figures 3G,H).

There were no differences between GC and FLT the germination rates of either Col-0 or *spr1*. The 4d harvest images (Figures 3A,B,E,F) were used to compare seedlings with any ungerminated seeds; germination in both genotypes was 100% under both GC and FLT conditions (Supplementary Table S2).

#### The *spr1* Spaceflight Response Involved Fewer Transcriptomic Changes Than Col-0

**FIGURE 4:**
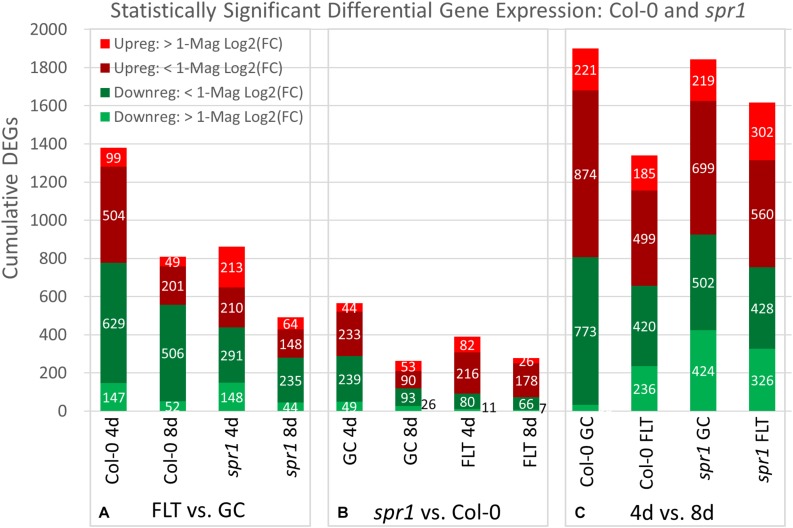
Col-0 and *spr1* overall differential gene expression levels. A graphic representation of the genes differentially expressed in the **(A)** FLT vs. GC, **(B)** 4d vs. 8d, and **(C)** *spr1* vs. Col-0 comparisons. Upregulation of a gene denotes a higher expression level in the first context of the comparison. Genes are separated into categories based on direction of differential expression and a Log_2_(Fold-Change) magnitude cut-off of one. d, day; DEG, differentially expressed gene; Downreg, downregulated; FLT, spaceflight; GC, ground control; Mag, magnitude; Upreg, upregulated. Data are based on four biological replicates.

Spaceflight-conditioned differential gene expression patterns were highly dependent upon the genotype, ecotype, and developmental age of seedlings. Quantitative gene expression data revealed patterns from three perspectives: spaceflight versus GCs for each genotype at each developmental age (Figure 4A: FLT vs. GC), wild type plants versus the mutant genotype in each environment and age (Figure 4B: *spr1* vs. Col-0) and between the two developmental ages (Figure 4C: 4d vs. 8d). The numbers of differentially expressed genes (DEGs) were categorized according to the magnitude of their fold-change values in the Col-0 and *spr1* roots. In the FLT vs. GC comparison (Figure 4A), both lines differentially expressed fewer genes at the 8d timepoint than at 4d in all fold-change categories. The spaceflight acclimation of *spr1* also required fewer genes than Col-0 to be differentially expressed. In the direct comparison of *spr1* with Col-0 (Figure 4B), more DEGs were observed within each growth condition at 4d than at 8d. The difference in total DEG count between conditions was also reduced at 8d. This occurred in almost all fold-change categories, and differences in total DEGs between the lines diminished between timepoints irrespective of the growth condition. In the developmental comparison of expression between the 4d and 8d timepoints (Figure 4C), the roots from the FLT context showed lower counts of total DEGs than their respective GC roots. However, while *spr1* required slightly fewer genes to be differentially expressed during development in the GC context, the opposite was true in FLT, where *spr1* required more DEGs for spaceflight development than Col-0. Twofold-change categories that did not follow these trends were those of the DEGs upregulated and downregulated more than twofold across *spr1* and Col-0 development, respectively, which were larger in FLT than in GC.

The relative contributions of DEGs with high degrees of differential expression (greater than twofold change) in response to spaceflight were variable among genotypes and developmental age. In the FLT vs. GC comparison (Figure 4A), the proportion of these DEGs was elevated in *spr1* (4d: 41.8%, 8d: 22.0%) relative to Col-0 (4d: 17.8%, 8d: 12.5%), and the difference in this proportion between the lines declined over time. However, when the genotypic comparison of s*pr1* vs. Col-0 (Figure 4B) was made, the proportion of these DEGs showed the opposite trend. In this case, the level of differential expression was slightly higher in FLT at 4d (GC: 16.5%, FLT: 23.9%) and at 8d the proportion of these DEGs was elevated in GC (GC: 30.2%, FLT: 11.9%). In the developmental comparison (Figure 4C), the primary point of interest was the low number of genes whose expression increased to a high degree over time in Col-0 GC roots (33 DEGs) when compared to the other contexts. In the same time frame, *spr1* increased expression of 424 genes over twofold to accomplish the same development.

#### *spr1* Differentially Expressed Defense Pathways in Its Spaceflight Response

**FIGURE 5:**
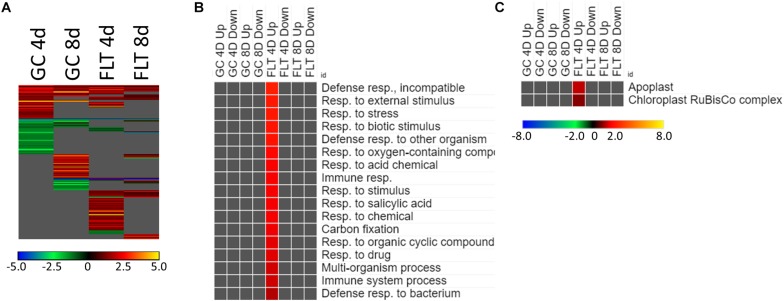
*Spr1* vs. Col-0; the effects of *SPR1* loss on gene expression. **(A)** Differentially expressed genes (DEGs) between the *spr1* mutant and its Col-0 wild-type background with greater than twofold magnitude in at least one context. DEG heatmaps are scaled such that yellow and blue represent more extreme levels of up- and downregulation, respectively, while red and green represent lower fold-changes in the same manner. In this case, upregulation of a gene indicates a higher expression level in *spr1* than in Col-0. **(B,C)** Gene ontology (GO) terms for **(B)** biological processes and **(C)** cellular compartments whose annotated genes were over- and underrepresented in the lists of DEGs for each context which met the twofold cutoff. The significance of the GO terms’ enrichment is represented by the –Log_10_ transform of its *q*-value. The scaling scheme used for the GO term heatmaps was similar to that described for DEGs, where yellow and red denote overrepresented terms, and blue and green denote underrepresented terms. Data are based on four biological replicates.

Most of the genes that are highly differentially expressed between the Col-0 and *spr1* are unique within the context of developmental age or growth environment (Figure 5A). However, a small number were differentially expressed between genotype irrespective of environment or age (Figure 5A). The coordinately expressed upregulated genes were related to the regulation of defense responses (NIMIN-1, ALD1, DLO1). The only genes that registered as coordinately downregulated in this comparison were SPIRAL1 and an antisense transcript (AT3G29644) of a transposable element which did not show differential expression itself.

The theme of stress response and defense was also evident in the GO enrichment analyses of the genes differentially expressed between Col-0 and *spr1* in the spaceflight environment. The significantly over- and underrepresented GO terms among biological processes are indicated in Figure 5B. Genes associated with general stress responses and environmental stimuli were prominent, as well as those annotated to terms related to defense responses. DEGs associated with these defense and stress pathways have been noted in the FLT vs. GC comparisons of previous studies (e.g., Correll et al., 2013; Paul et al., 2013, 2017; Choi et al., 2019). Genes involved in responses to salicylic acid, a phytohormone linked to both plant defense signaling and regulation of plant development (Klessig et al., 2018), were also enriched. Carbon fixation pathways, and specifically multiple ribulose bisphosphate carboxylase (RuBisCo) genes, were also upregulated in 4d FLT *spr1* (Figures 5B,C). Genes encoding proteins localized to the apoplast were also enriched among those upregulated in FLT *spr1*, indicating that significant remodeling of processes within the cell was also needed by *spr1* within the spaceflight environment (Figure 5C).

**FIGURE 6:**
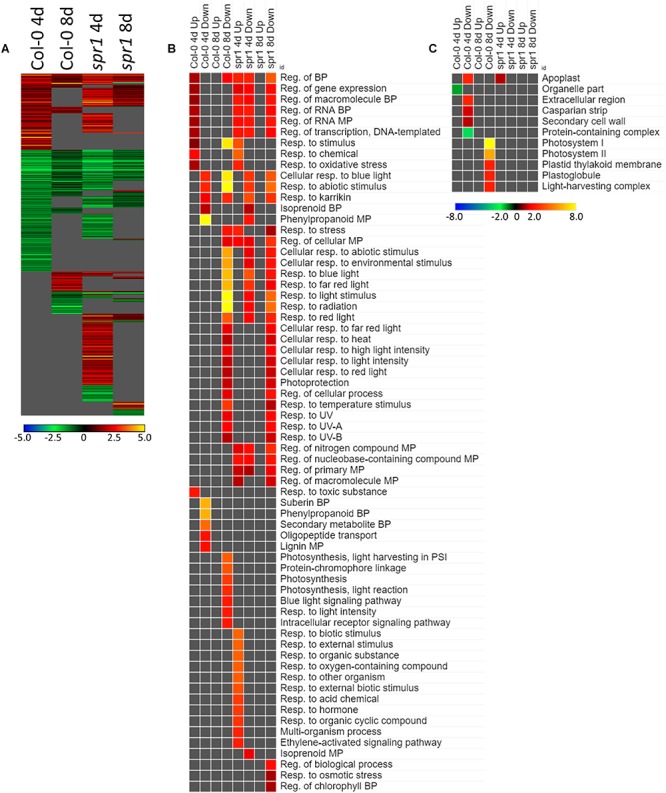
FLT vs. GC for Col-0 and *spr1*; the DEGs involved in spaceflight acclimation. **(A)** Differentially expressed genes (DEGs) between the GC and FLT conditions with greater than twofold magnitude in at least one context. DEG heatmaps are scaled such that yellow and blue represent more extreme levels of up- and downregulation, respectively, while red and green represent lower fold-changes in the same manner. In this case, upregulation of a gene indicates a higher expression level in FLT than the GC. **(B,C)** Gene ontology (GO) terms for **(B)** biological processes and **(C)** cellular compartments whose annotated genes were over- and underrepresented in the lists of DEGs for each context which met the twofold cutoff. The significance of the GO terms’ enrichment is represented by the –Log_10_ transform of its *q*-value. The scaling scheme used for the GO term heatmaps was similar to that described for DEGs, where yellow and red denote overrepresented terms, and blue and green denote underrepresented terms. Data are based on four biological replicates.

Many of the DEGs associated with the acclimation to spaceflight were unique to genotype and to the developmental age of the plants (Figure 6A). There were 554 genes differentially expressed by at least twofold between spaceflight and ground among the two age groups (4d and 8d) of the two genotypes (Col-0 and *spr1*), with 63 genes coordinately expressed across all plants. Developmental age had a substantial impact on the spaceflight response of both genotypes; in both Col-0 and spr1, the 4d plants had a higher number of DEGs than the 8d plants. There was a large amount of overlap between the GO processes identified from the DEGs used to acclimate to the FLT condition for *spr1* and Col-0 (Figure 6B). Among the more widely shared GO categories in the 4d plants of both genotypes were those corresponding to the regulation of gene expression and transcriptional processes. These were more highly enriched in *spr1* roots than in Col-0, however, it can also be seen that there is a *spr1*-specific enrichment of the same terms among those genes which are downregulated in the response to FLT. The term for a response to karrikin compounds (phytohormone-like molecules derived from burning plant material) was also indicated. Many light signaling terms also appeared as enriched among DEGs downregulated in FLT, and these were involved with the response to various wavelengths of light as well as the response to elevated light intensity. However, specific photosynthetic terms appeared amongst those which were downregulated only at 8d in Col-0. Similar localization terms appeared specifically in 8d Col-0 as well (Figure 6C), indicating that these were changes in the expression of genes directly involved in the photosynthetic machinery. While both *spr1* and Col-0 4d were enriched with DEGs localized to the apoplast, these were among the upregulated genes in *spr1* and the downregulated genes in Col-0, alongside other terms related to extracellular structures. Furthermore, terms related to organellar localization were significantly underrepresented in those genes upregulated in Col-0 at 4d.

**FIGURE 7:**
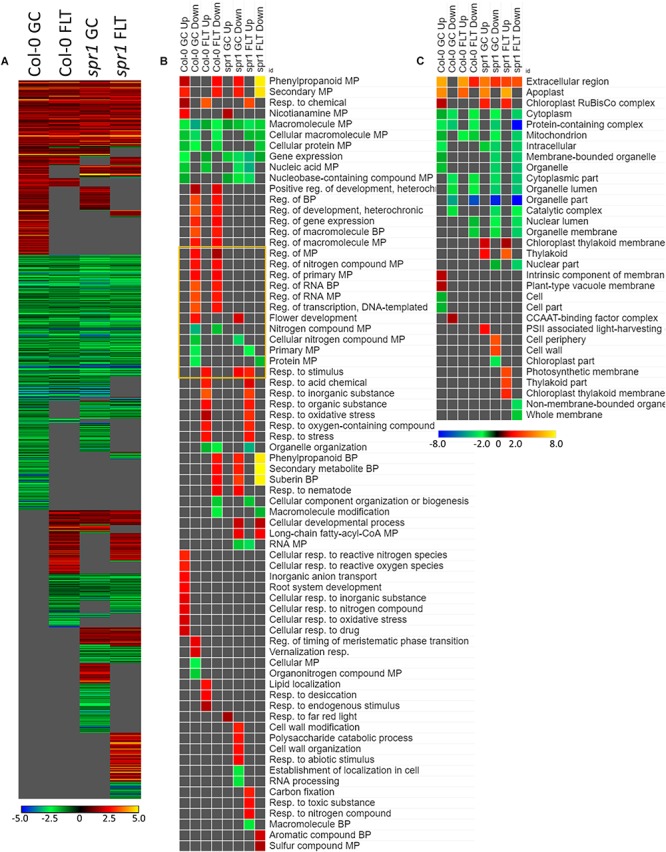
4d vs. 8d seedlings of Col-0 and *spr1*; developmentally-associated DEGs. **(A)** Differentially expressed genes (DEGs) between the 4d and 8d timepoints with greater than twofold magnitude in at least one context. DEG heatmaps are scaled such that yellow and blue represent more extreme levels of up- and downregulation, respectively, while red and green represent lower fold-changes in the same manner. In this case, upregulation of a gene indicates a higher expression level at 4d than at 8d. **(B,C)** Gene ontology (GO) terms for **(B)** biological processes and **(C)** cellular compartments whose annotated genes were over- and underrepresented in the lists of DEGs for each context which met the twofold cutoff. The significance of the GO terms’ enrichment is represented by the –Log_10_ transform of its *q*-value. The scaling scheme used for the GO term heatmaps was similar to that described for DEGs, where yellow and red denote overrepresented terms, and blue and green denote underrepresented terms. Data are based on four biological replicates.

The expression of genes associated with the developmental age was impacted by the spaceflight environment (Figure 7A). Approximately 26% of the total DEGs meeting the criteria for inclusion were required to be differentially expressed at some level regardless of the growth condition or genotypic background to facilitate development. However, among only those DEGs which met the twofold change cutoff, there were not GO terms shared so ubiquitously between contexts (Figure 7B). While broad GO terms for gene expression and metabolic processes were underrepresented in many of the contexts, more specific terms related to developmental and metabolic regulation were enriched during development in Col-0. Genes related to oxidative stress were also enriched among those genes more highly expressed at 4d in FLT in both genotypes. Localization GO term enrichments were highly divided between intracellular and extracellular compartments, which were consistently underrepresented and overrepresented, respectively (Figure 7C). These underrepresented GO terms occur mostly within the sets of DEGs which were upregulated between 4d and 8d, with many more significantly enriched in *spr1*. The primary exception to this was a set of terms related to the chloroplast and the RuBisCo complex, which were mostly enriched in those DEGs more highly expressed in early development of *spr1*.

### WS and *Sku5*

#### Germination and Morphology of *sku5* in Spaceflight

**FIGURE 8:**
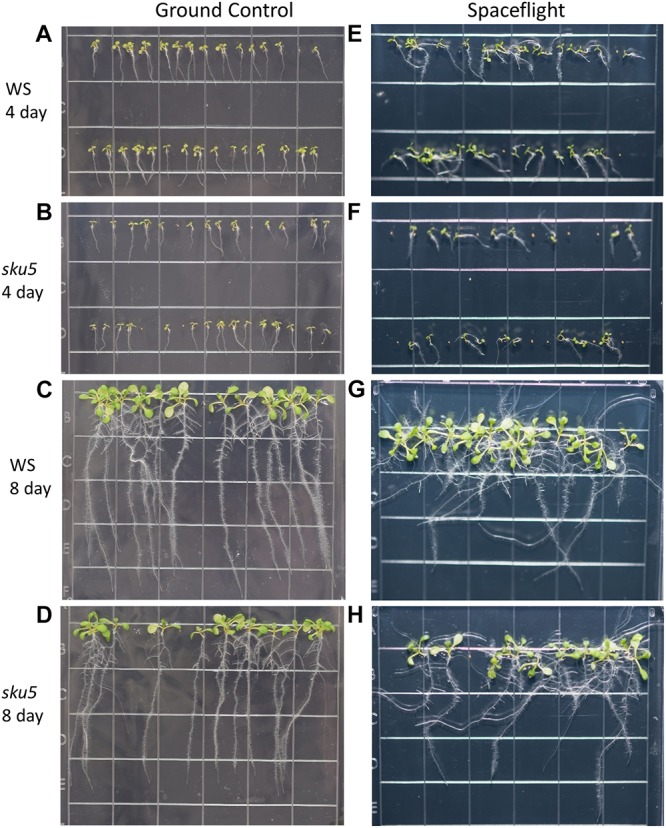
High resolution images of the WS and *sku5* growth patterns. Images are arranged such that the ground control seedlings **(A–D)** are in the left column while spaceflight seedlings are on the right **(E–H)**. Vertically, images alternate between WS **(A,C,E,G)** and *sku5* **(B,D,F,H)**. The images taken at the 4d time point **(A,B,E,F)** are above those taken at the 8d time point **(C,D,G,H)**. Images taken from above the growth medium at the time of harvest. Grid squares are 13 mm wide.

Phenotypic differences among the WS background plants in GC and FLT environments included root length and skewing angles (Figures 2, 8). The primary root lengths of FLT WS were decreased significantly compared to GC at 8d (two-tailed *t-*test, *p* = 0.000209), but not at 4d. There were no differences in root lengths between GC and FLT *sku5* plants of either 4d or 8d plants (Figures 2B, 8C,D,G,H). There were differences in root length between WS and *sku5* genotypes in the GC (*p* = 0.0013) but FLT WS and FLT *sku5* roots were not statistically different (Figures 2B, 8C,D,G,H).

There were no statistically significant differences in the overall change from vertical between WS and *sku5* in either GC or FLT environments (Figures 2C, 8A–D). The differences in root angles between GC and FLT for each genotype, and between the WS and *sku5* genotypes in both FLT and GC environments, also failed to meet statistical criteria. However, visual inspection of the images, and also the plots of Figure 2C, illustrate the difference in the directional trends for root growth in each genotype and environment. For both genotypes the variance in the growth angle (as noted in the SD values in Figure 2) was greater in the spaceflight samples.

The FLT *sku5* seeds germinated at a significantly reduced rate (48%) compared to WS FLT (85%), and to *sku5* GC (77%) and WS GC (93%) [χ^2^ (3, *N* = 185) = 15.01, *p* = 0.00181] (Supplementary Table S2 and Figures 8A,B,E,F). However, the FLT *sku5* seeds that germinated produced plants that were comparable to their GC counterparts, as evidenced both by plate images and by the lack of difference in root length between FLT and GC *sku5* at 4 and 8d (Figures 2B, 8B,D,F,H).

#### The Expression of More Genes Is Altered in *sku5* Than in WS During Spaceflight

**FIGURE 9:**
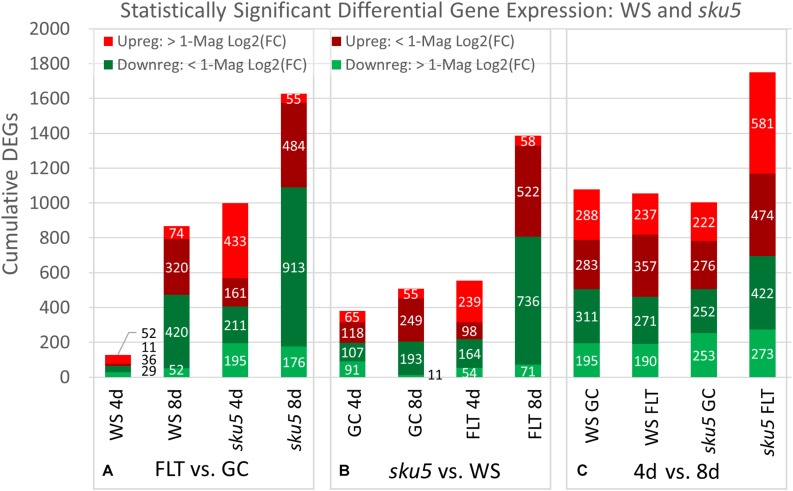
WS and *sku5* overall differential gene expression levels. A graphic representation of the genes differentially expressed in the **(A)** FLT vs. GC, **(B)** *sku*5 vs. WS, and **(C)** 4d vs. 8d comparisons. Upregulation of a gene denotes a higher expression level in the first context of the comparison. Genes are separated into categories based on direction of differential expression and a Log2(Fold-Change) magnitude cut-off of one. d, day; DEG, differentially expressed gene; Downreg, downregulated; FLT, spaceflight; GC, ground control; Mag, magnitude; Upreg, upregulated. Data are based on four biological replicates.

The trends of differential expression in WS and the *sku5* line contrasted with those seen in Col-0 and *spr1* in that WS and *sku5* show an elevated transcriptomic response to spaceflight over time, with *sku5* further differentially expressing more genes than WS (Figure 9). The clearest example of this was the FLT vs. GC comparison of gene expression for these lines, where the number of DEGs required for spaceflight acclimation of *sku5* were fivefold and twofold more than WS at 4d and 8d, respectively (Figure 9A: FLT vs. GC). However, both genotypes increase in their DEG counts between 4d and 8d. The proportion of DEGs with a greater than twofold change also decreased to a very similar degree for both WS (4d: 63.2%, 8d: 14.5%) and *sku5* (4d: 62.8%, 8d: 14.2%) despite the large difference in counts. When the genotypes were compared directly, the transcriptomic differences between them were exacerbated as they acclimated to the flight condition over time (Figure 9B: *sku5* vs. WS). The proportion of DEGs meeting the twofold change criteria followed a similar trend to that seen in flight acclimation, where it decreased in both GC (4d: 40.9%, 8d: 13.0%) and FLT (4d: 52.8%, 8d: 9.3%). Despite these trends, most genes among the FLT 4d DEGs that had greater than twofold change were those that were expressed more highly in *sku5*. This trend persisted into the 4d vs. 8d comparison (Figure 9C: 4d vs. 8d), where *sku5* in the FLT condition required more genes to be differentially expressed in order to develop. S*ku5* in the GC condition behaved much like WS in both FLT and GC conditions in terms of DEG counts in each fold-change category. Together, these patterns of gene expression indicated that the conditions of spaceflight had a disproportionate effect on the *sku5* roots’ transcriptomic responses, but that each genotype’s response to spaceflight over time involved a greater number of DEGs of lower fold-changes.

#### *Sku5* Highly Induced ABA- and Stress-Associated Genes in Spaceflight

**FIGURE 10:**
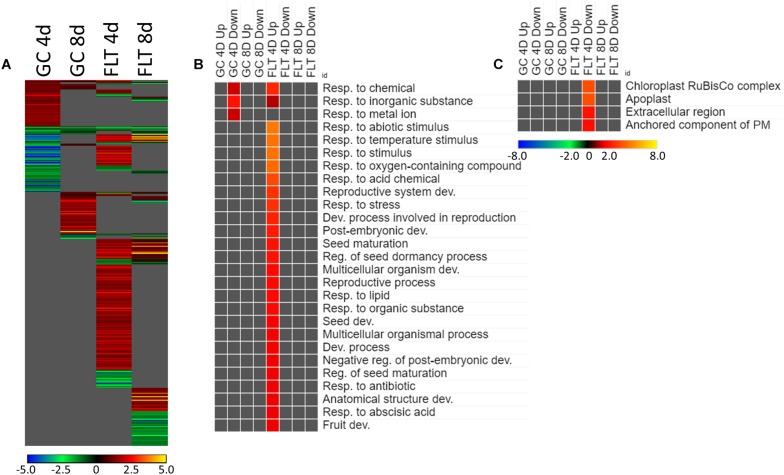
*sku5* vs. WS; the effects of *SKU5* loss on gene expression. **(A)** Differentially expressed genes (DEGs) between the *sku5* mutant and its WS wild-type background with greater than twofold magnitude in at least one context. DEG heatmaps are scaled such that yellow and blue represent more extreme levels of up- and downregulation, respectively, while red and green represent lower fold-changes in the same manner. In this case, upregulation of a gene indicates a higher expression level in *sku5* than in WS. **(B,C)** Gene ontology (GO) terms for **(B)** biological processes and **(C)** cellular compartments whose annotated genes were over- and underrepresented in the lists of DEGs for each context which met the twofold cutoff. The significance of the GO terms’ enrichment is represented by the –Log_10_ transform of its *q*-value. The scaling scheme used for the GO term heatmaps was similar to that described for DEGs, where yellow and red denote overrepresented terms, and blue and green denote underrepresented terms. Data are based on four biological replicates.

The genes differentially expressed between *sku5* and WS were similar between GC and FLT growth conditions (Figure 10A), in that the same genes that were highly upregulated in WS GC were highly upregulated in FLT for *sku5*. Among these were many late embryogenesis abundant (LEA) family proteins, whose expression has been connected to enhanced resistance to abiotic stresses and alterations in ABA sensitivity (Zhao et al., 2011; Candat et al., 2014). Additional LEA family genes and seed storage proteins, such as CRUCIFERINA (CRA1) and CRUCIFERIN 3 (CRA3) were seen as upregulated uniquely in *sku5* FLT. Alongside these were further genes associated with ABA- or ABA-independent dormancy signaling, notably REDUCED DORMANCY 5 (RDO5), DELAY OF GERMINATION 1 (DOG1), and HIGHLY ABA-INDUCED PROTEIN 2 and 3 (HAI2, HAI3). In 4d *sku5* spaceflight plants, genes encoding LEAs and other seed-associated proteins are among the most highly induced (Log_2_[5 to 10], or 30 to 1000-fold). Many of these patterns are further supported by GO biological process term enrichments (Figure 10B), where almost all of the overrepresented terms are enriched within the group of genes more highly expressed by *sku5* roots at 4d in FLT. Outside of the mentioned ABA signaling and seed development genes, terms for regulation of seed dormancy and negative regulation of post-embryonic development are overrepresented, alongside more general terms such as responses to temperature and stress. All of the significant GO localization enrichments were for genes more highly expressed in WS in the 4d FLT context, involving either DEGs related to the RuBisCo complex or extracellular structures (Figure 10C). DEGs encoding proteins anchored to the PM are enriched for this group, indicating an important role for them in WS.

**FIGURE 11:**
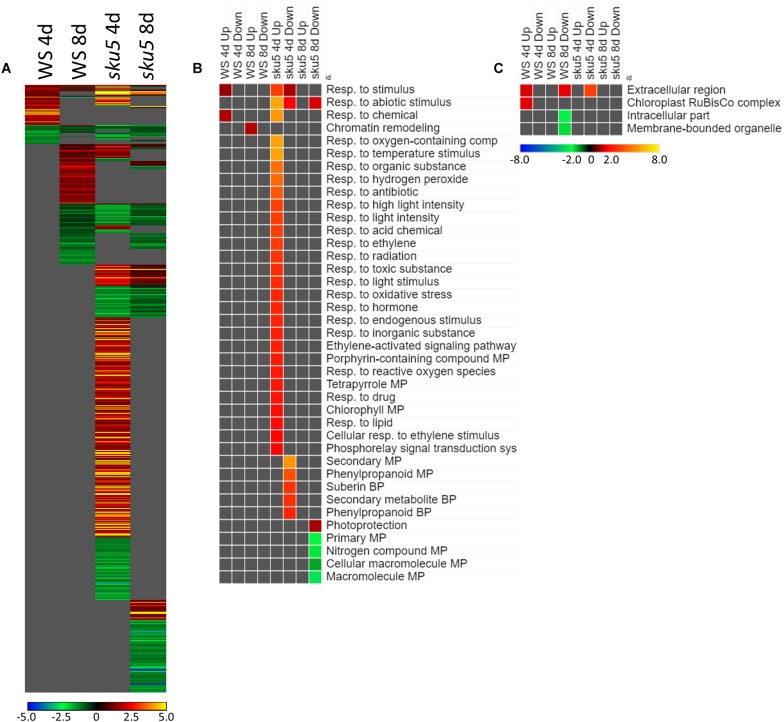
FLT vs. GC for WS and *sku5*; DEGs involved in spaceflight acclimation. **(A)** Differentially expressed genes (DEGs) between the GC and FLT conditions with greater than twofold magnitude in at least one context. DEG heatmaps are scaled such that yellow and blue represent more extreme levels of up- and downregulation, respectively, while red and green represent lower fold-changes in the same manner. In this case, upregulation of a gene indicates a higher expression level in FLT than the GC. **(B,C)** Gene ontology (GO) terms for b biological processes and **(C)** cellular compartments whose annotated genes were over- and underrepresented in the lists of DEGs for each context which met the twofold cutoff. The significance of the GO terms’ enrichment is represented by the –Log_10_ transform of its *q*-value. The scaling scheme used for the GO term heatmaps was similar to that described for DEGs, where yellow and red denote overrepresented terms, and blue and green denote underrepresented terms. Data are based on four biological replicates.

Many of the LEAs and ABA-responsive DEGs in the *sku5* vs. WS comparison were also highly upregulated by *sku5* in the FLT vs. GC comparison, and some were unique to the spaceflight acclimation of *sku5* (Figures 10A, 11A). The majority of FLT vs. GC DEGs (70.3%) were only detected in one of the *sku5* FLT vs. GC comparison contexts. As such, the majority of the GO biological process terms found to be enriched were unique to the DEGs upregulated in FLT *sku5* at the 4d time point (Figure 11B). Hormone response and signal transduction terms were enriched, with ethylene signaling, ROS response, and light signaling among the more specific pathways to be represented. In tandem, these 4d *sku5* roots had decreased enrichment of DEGs associated with the synthesis of suberin. Uniquely to this comparison, 8d FLT WS roots upregulated genes related to chromatin remodeling. As seen in other comparisons (Figures 6C, 7C, 10C), WS upregulated genes annotated to the RuBisCo complex in FLT (Figure 11C). Additionally, the 8d downregulated DEGs for WS were overrepresented for extracellular localization, and DEGs whose products localized to intracellular and organellar compartments were underrepresented (Figure 11C). However, *sku5* showed overrepresentation of downregulated DEGs annotated to the extracellular region at the earlier time point of 4d.

**FIGURE 12:**
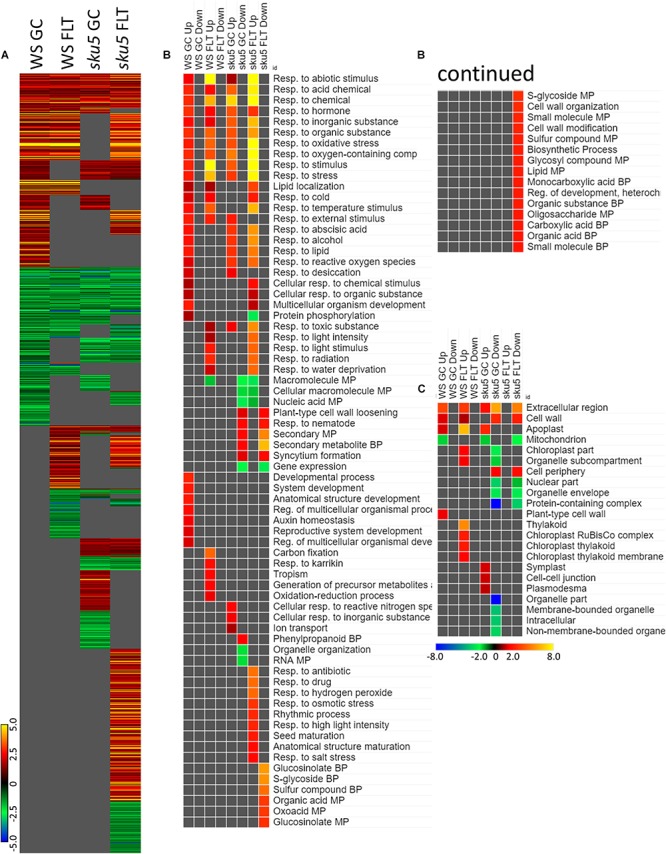
4d vs. 8d seedlings of WS and *sku5*; developmentally-associated DEGs. **(A)** Differentially expressed genes (DEGs) between the 4d and 8d timepoints with greater than twofold magnitude in at least one context. DEG heatmaps are scaled such that yellow and blue represent more extreme levels of up- and downregulation, respectively, while red and green represent lower fold-changes in the same manner. In this case, upregulation of a gene indicates a higher expression level at 4d than at 8d. **(B,C)** Gene ontology (GO) terms for **(B)** biological processes and **(C)** cellular compartments whose annotated genes were over- and underrepresented in the lists of DEGs for each context which met the twofold cutoff. The significance of the GO terms’ enrichment is represented by the –Log_10_ transform of its *q*-value. The scaling scheme used for the GO term heatmaps was similar to that described for DEGs, where yellow and red denote overrepresented terms, and blue and green denote underrepresented terms. Data are based on four biological replicates.

Late embryogenesis abundant family genes were also prominently represented in the 4d vs. 8d comparison within GC and FLT environments (Figure 12A). Examination of the GO biological process terms yielded by the individual lists of DEGs showed many similar stress response terms, but these were primarily in the context of genes more highly expressed at the 4d time point (Figure 12B). However, based on the level of significance of the terms, the enrichment of DEGs related to many of these stress responses was enhanced in FLT. In the cases of oxidative stress and cold response *sku5* showed this enhancement. However, in some cases, such as the response to desiccation, both genotypes showed a lack of significant term enrichment in FLT. The *sku5* roots also showed slightly higher significance of enrichment of DEGs associated with responses to water deprivation and light stimuli in early FLT development, compared to WS. GO terms related to cell wall remodeling and immune responses, commonly seen in FLT acclimation, were also enhanced in their enrichment during FLT development of *sku5* compared to GC development. The remainder of the significant GO terms were unique to one context, and among these is the response to karrikin, which was previously seen in the context of *spr1* and Col-0 FLT vs. GC comparisons (Figure 6B). Of the terms enriched specifically in *sku5* FLT development, processes involved with salt and ROS stress, as well as light responses, were significantly higher at 4d. At 8d, the terms associated with *sku5* in FLT were primarily focused on cell wall remodeling and biosynthetic processes.

## Discussion

The discovery that root skewing was not gravity-dependent, and prevalent in both light-grown and dark-grown plants in spaceflight habitats (Millar et al., 2011; Paul et al., 2012a; Nakashima et al., 2014), invited questions about the underlying mechanisms of this behavior in the microgravity environment and the relationship between those mechanisms and physiological adaptation to spaceflight. The experiments described here examined the contribution of two well-characterized skewing genes that are in two different ecotypic backgrounds and which affect different skewing control pathways. SPIRAL1 plays a role in directional cell expansion by regulating cortical microtubule dynamics (Furutani et al., 2000; Nakajima et al., 2004; Sedbrook et al., 2004; Galva et al., 2014). SKU5 is a skewing-related glycosylphosphatidylinositol-anchored cell wall and plasma membrane protein, which has also been implicated in stress response signaling (Sedbrook et al., 2002). The *sku5* and *spr1* mutants are well characterized in terrestrial environments, skewing rightward and leftward, respectively, when grown vertically (Furutani et al., 2000; Sedbrook et al., 2002, 2004; Nakajima et al., 2004). The experimental readouts used to assess the physiological adjustment of seedlings to spaceflight were root and germination measurements, and the differences in transcriptomic responses among the genotypes. The opposing directions of the skewing phenotypes of the mutant lines are believed to stem from alterations of distinct cellular processes, and this is most clearly evidenced by the differential effects of microtubule-destabilizing agents on these mutants (Furutani et al., 2000; Sedbrook et al., 2002, 2004; Nakajima et al., 2004; Galva et al., 2014). The distinct roles of these two genes in determining root directionality is supported by their different individual responses to spaceflight in the present experiments. The data from *sku5* and *spiral1* are analyzed in context with their parent genotype, WS and Col-0 respectively, as WS shows a much smaller number of spaceflight differentially expressed genes than Col-0 (Paul et al., 2017, and Figures 4, 12).

### *Spr1* Skews Leftward on Earth and Moreso in Space

Terrestrial studies concluded that SPR1 reinforces rightward root growth by affecting the balance of thigmotropism and gravitropism (Galva et al., 2014). Morphological data from APEX-03-2 support this notion. At the 4d time point, *spr1* seedlings showed a consistent leftward skewing phenotype on Earth, and the leftward skewing was substantially enhanced by spaceflight (Figures 2C, 3B,F). However, *spr1* and Col-0 roots were the same length in spaceflight, indicating that the role of SPR1 lies in directionality rather than overall root length in spaceflight (Figure 2A). These data suggest that in the absence of gravitropism during spaceflight, thigmotropism plays a dominant role in growth directionality and that SPR1 acts to positively enhance skewing while not affecting root length in spaceflight. The spaceflight effect of *spr1* lies in morphology management rather than growth management.

SPR1 binds with another microtubule plus-end tracking protein, EB1b (Galva et al., 2014). It has been postulated that EB1b and SPR1 work in concert to regulate mechanical force-based directional growth with a balance of gravitropism and thigmotropism, where SPR1 participates in the reinforcement of rightward growth through thigmotropic processes, while EB1b negatively regulates thigmotropism and positively regulates gravitropism (Galva et al., 2014). In an environment lacking a gravity cue such as spaceflight, this gravitropic reinforcement has no effect, resulting in the enhanced skewing of *spr1.* Similarly elevated leftward skewing of *spr1* occurs under cold conditions alongside alterations of the microtubule network, and skewing is conversely suppressed by heat or salt treatments (Furutani et al., 2000; Sedbrook et al., 2004). The elevated skewing seen in *spr1* may therefore be the result of spaceflight-induced signals in the root which are similar to those involved in cold response signaling. The microtubule-associated protein MAP65-1 is more abundant in spaceflight, and is also hypothesized to be linked to cold-responsive microtubule stabilization (Chen et al., 2016; Soga et al., 2018). Actin cytoskeleton mutants also exhibit a greater degree of skewing in microgravity when compared to wild-type and GC plants, though this skewing occurs to the right when regarded from behind the media (Nakashima et al., 2014). These structural systems both appear to have roles in suppressing endogenous root skewing patterns in spaceflight. The spaceflight-enhanced skewing of *spr1* may therefore be attributed to its altered microtubule dynamics in the spaceflight response, similar to altered dynamics induced by terrestrial cold stress.

### *Sku5* Skews Inappropriately in Spaceflight

The *sku5* mutant did not show an enhancement of skewing in spaceflight as compared to the GC, nor did *sku5* respond to the spaceflight environment in the same manner as it does to terrestrial disruption of the gravity vector (Figure 2C) (Sedbrook et al., 2002). However, *sku5* and WS roots were the same length in spaceflight, indicating that the role of SKU5 also lies in directionality rather than overall root length in spaceflight (Figure 2B). These data suggest that SKU5, like SPR1, regulates morphology and directionality rather than growth. However, SKU5 regulation is very different from SPR1 regulation of skewing morphology in that lack of SKU5 function prevents appropriate skewing in spaceflight. This prevention of skewing, the *sku5* interruption of the pathways involved in skewing, leads to dramatic changes in differential expression of genes for physiological adaptation to spaceflight.

### Differential Gene Expression as a Measure of the Quantity and Character of the Physiological Adaptation to Spaceflight

Differential gene expression profiles reveal the underlying gene expression changes needed for physiological adaptation to environmental changes. There are numerous, well-documented environmentally-induced gene expression profiles in plants, all of which detail specific physiological changes related to the specific stresses. The intensity of the differential gene expression response, in terms of the number of genes involved and their fold-change levels, may be considered a measure of the metabolic cost of adapting to that environment. A terrestrial example of this metabolic cost is in the transcriptomic response to cold stress in Arabidopsis lines that are either sensitive or resistant to cold (Chan et al., 2016). Plants that overexpressed RNA-DIRECTED DNA METHYLATION 4 (RDM4) had a cold-tolerant phenotype, and exhibited a greatly reduced transcriptomic response to cold stress compared to the more cold-sensitive wild-type and *rdm4* knock-out plants (Chan et al., 2016). The plants that were least able to tolerate cold environments displayed a far higher number (up to 100×) of differentially expressed genes in response to cold than the cold-tolerant plants exposed to the same environment (Chan et al., 2016).

*Spr1* adapts to spaceflight with far fewer DEGs than Col-0. This suggests that mutation of the SPR1 skewing pathway enhances spaceflight physiological adaptation. In contrast, the *sku5* mutation results in a doubling of DEGs in response to spaceflight, and many of those DEGs are members of strong environmental stress responses. This strong response suggests that the SKU5 pathway plays a key role in spaceflight physiological adaptation.

#### *Sku5* Mutation Produces a Dramatic Increase in Differential Gene Expression in Spaceflight

**FIGURE 13:**
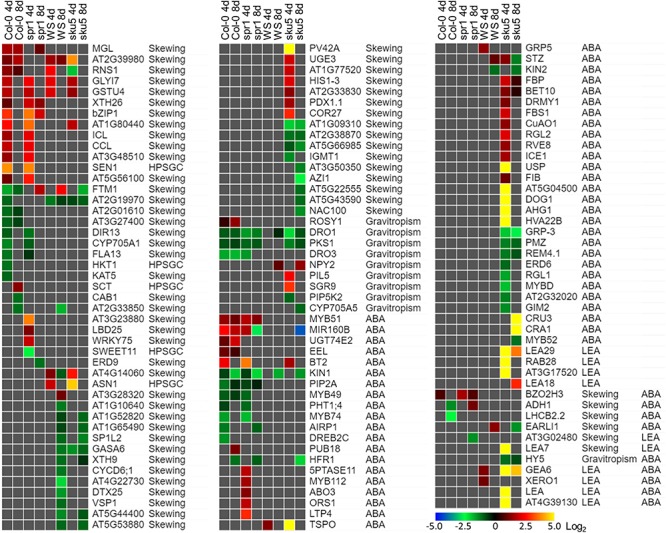
Differentially expressed genes between FLT and GC of particular interest. Statistically significant DEGs with greater than twofold magnitude in at least one context. Each gene in this list corresponded to the following categories: potential and highly probable skew gene candidates (Skewing, HPSGC; Schultz et al., 2017), late embryogenesis abundant (LEA; Hundertmark and Hincha, 2008), associated with gravitropism (GO Terms; Carbon et al., 2008), and abscisic acid (ABA, GO Terms; Carbon et al., 2008). The heat map scale Log_2_(Fold-Change) is indicated in the bottom right corner. Categorical information is provided beside each gene group. Data are based on four biological replicates.

*Sku5* showed a dramatic program of differential gene expression compared to the GCs, a program that was more pronounced and diverse than that of *spr1*. Many of the genes that are significantly differentially expressed between spaceflight and GCs are associated with skewing and cell wall remodeling, irrespective of intensity of skewing phenotype in the spaceflight-grown plants (Figure 13).

One of the unique aspects of the *sku5* spaceflight differential gene expression program involved genes usually associated with dehydration and ABA signaling. The FLT *sku5* expression patterns of LEAs and other ABA-associated genes suggest a spaceflight adaptation phenomenon involving, but not limited to, an altered ABA sensitivity in these plants. Many of the highest magnitude DEGs in the spaceflight acclimation of *sku5* were in the LEA family, primarily dehydrins and group-4 LEAs; many of these genes were induced by greater than 30-fold [Log_2_(5)] (Figure 13). LEAs are responsive to ABA signaling resulting from osmotic, cold, and drought stresses and act to enhance stress resistance through membrane stabilization, sequestration of ROS, and prevention of protein aggregation (Hundertmark and Hincha, 2008; Zhao et al., 2011; Candat et al., 2014; Fang and Xiong, 2015; Suzuki et al., 2016).

Many of the DEGs and LEAs upregulated in the spaceflight acclimation of *sku5* are associated with embryonic programs that are adjusted at the chromatin level during germination in response to environmental factors (Tai et al., 2005; Tang et al., 2012; Molitor et al., 2014). DOG1, for example, increases in expression in cold temperatures, acting to repress germination during those unfavorable conditions (Footitt et al., 2013, 2014). The *sku5* DEGs HAI2 and HAI3 are involved in regulating ABA signaling and responding to soil water potential (Bhaskara et al., 2012; Kim et al., 2013). REDUCED DORMANCY 5 also acts to inhibit germination, but functions independently of DOG1 and ABA signaling pathways (Xiang et al., 2014). The GO term enrichments for *sku5* also support this, as DEGs associated with dormancy regulation are overrepresented in the dataset (Figures 10B, 12B). It is important to note that SKU5 is expressed in floral tissue (Sedbrook et al., 2002). This altered ABA signaling in *sku5* could therefore be attributed to downstream effects of altered seed development. In support of these interpretations, germination rates were repressed in FLT *sku5* (Figures 8F,H) relative to GC *sku5* (Figures 8B,D), and to FLT WS (Figures 8E,G), as evidenced by counts of ungerminated seeds in the harvest images (Supplementary Table S2). While *spr1* is also expressed in reproductive tissues, the differential expression of dormancy signaling genes was not prevalent in the Col-0 and *spr1* comparisons (Nakajima et al., 2004; Sedbrook et al., 2004). In fact, those few DEGs related to ABA signaling observed in Col-0 and *spr1* displayed a trend in expression that was opposite of what was seen in FLT *sku5*. As a prime example, the LEA ABA-RESPONSE PROTEIN (ABR) was only slightly downregulated in the spaceflight acclimation of 8d *spr1*. ABA-related dormancy signaling may be affected by the spaceflight environment.

Differential expression patterns also indicate that *sku5* mutants experience changes in the plasma membrane during spaceflight. Alterations in plasma membrane dynamics and composition occur in plant responses to terrestrial stressors (e.g., cold stress) and in the response of *Chlorella vulgaris* to spaceflight (Popova et al., 1989; Solanke and Sharma, 2008; Kazemi-Shahandashti and Maali-Amiri, 2018). Roots of *Pisum sativum* grown under clinorotation also show shifts in membrane composition in sterol-enriched membrane domains known as “lipid rafts” (Kordyum et al., 2018). Membrane-bound GPI-APs, such as SKU5, localize to these lipid raft nanodomains and are important to stress acclimation, including spaceflight-associated stress (Minami et al., 2008; Mazars et al., 2014; Ferl et al., 2015; Takahashi et al., 2016, 2019; Yeats et al., 2018). ABA signaling proteins also localize to these membrane domains in a sterol-dependent manner (Demir et al., 2013). Given that *sku5* shows alterations of ABA signaling pathways in spaceflight (Figure 13), it can be hypothesized that the lack of SKU5 may affect the function of these nanodomain-localized ABA signaling processes under stress conditions. The expression of a chloroplastic aldehyde reductase, ChlADR (AT1G54870), involved in the detoxification of reactive carbonyls formed as a result of lipid peroxidation, and TSPO, which participates in mobilization of lipids, are induced by stress and highly induced in the spaceflight acclimation of *sku5* (Guillaumot et al., 2009; Yamauchi et al., 2011; Jurkiewicz et al., 2018). These patterns match the gene expression trends of the spaceflight-induced LEA family genes that are thought to directly participate in membrane stabilization under stress (Battaglia et al., 2008; Hundertmark and Hincha, 2008; Candat et al., 2014). The gene expression patterns of these ABA-responsive genes in *sku5* suggest that SKU5 affects ABA signaling in response to stresses. Furthermore, this shows that pathways affecting membrane stabilization and remodeling are required for the spaceflight acclimation of *sku5* that are not essential for its growth in terrestrial environments.

SKU5 may also directly participate in stress response signaling or cellular remodeling due to its localization to the outer aspects of the plasma membrane. A SKU5 homolog is highly upregulated [Log_2_(14)] in tobacco overexpressing a FATTY ACID DESATURASE 3 (CbFAD3-OE) homolog derived from the cryophyte *Chorispora bungeana* when compared to a wild-type line in the response to salt stress (Shi et al., 2018). CbFAD3-OE lines exhibited enhanced membrane fluidity and survival under cold, salt, and drought stresses, and demonstrated reduced lipid peroxidation and membrane leakage via activation of calcium signaling and suppression of ROS (Shi et al., 2018). As such, the *sku5* line may require alternative pathways to be activated to maintain membrane fluidity and integrity during spaceflight acclimation due to the removal of SKU5 from this response pathway. The transcriptomic response of *sku5* to the spaceflight condition was elevated compared to WS, which would support the activation of additional pathways (Figures 10A, 11A). ROS-related genes are abundant in spaceflight transcriptomes, and reflect a response to spaceflight that is conserved among Arabidopsis cultivars and related species (Paul et al., 2013, 2017; Sugimoto et al., 2014; Ferl et al., 2015; Sng et al., 2018; Choi et al., 2019; Zhou et al., 2019). Our findings were consistent with this trend across genotypes and the skewing mutants based on GO term enrichments associated with the spaceflight response (Figures 6B, 11B). Notably, *sku5* FLT showed elevated expression of genes required to be expressed in response to ROS at the 4d when compared to FLT WS (Figures 9B, 10B), supporting this hypothesized function of SKU5 in this membrane-associated ROS suppression. While FAD3 was not among the *sku5* FLT DEGs, FLORAL TRANSITION AT THE MERISTEM1 (FTM1), which catalyzes the desaturation of stearic acid to oleic acid upstream of FAD3, was upregulated in WS and *spr1* spaceflight acclimation, and downregulated in that of Col-0 and *sku5.* FTM1 participates alongside FAD3 in the mediation of stresses associated with Arabidopsis crown gall development through their enrichment with C18:3 fatty acids (Klinkenberg et al., 2014). The increase of C18:3 class of fatty acids also correlated with the enhanced membrane stability measures in the CbFAD3-OE lines (Shi et al., 2018). These results therefore suggest a role for SKU5 in stress metabolism with respect to the plasma membrane, a role that was not revealed in previous ground experiments.

An additional explanation for the altered spaceflight response of *sku5* comes from its likely association with auxin metabolism and signaling upstream of the plant TOR complex. A SKU5 homolog in maize encodes an interactor of AUXIN-BINDNG PROTEIN 1 (ABP1) (Shimomura, 2006). The *abp1* mutant demonstrates a leftward-skewing phenotype opposite to that seen in *sku5* (Sedbrook et al., 2002; Gao et al., 2015). Auxin gradients are still present in spaceflight root tips despite the lack of a gravity cue in the spaceflight environment (Ferl and Paul, 2016). The presence of SKU5 acting to mediate anisotropic cell growth in response to endogenous auxin gradients could facilitate the spaceflight acclimation of wild-type seedlings. GO terms associated with the DEGs of 4d spaceflight response of *sku5* suggested this connection as well, with many alterations occurring in signaling pathways related to ethylene, ROS, and light signaling (Figure 11B). Furthermore, ABP1 is associated with the activation of TOR and the promotion of growth through the action of an auxin-activated signaling pathway mediated by the RHO OF PLANTS 2 (ROP2) GTPase (Xu et al., 2014; Schepetilnikov et al., 2017; Ryabova et al., 2019). The TOR complex integrates diverse environmental signals to regulate growth, and ABA signaling inhibits TOR, promoting autophagy to allow for stress tolerance acquisition (Ryabova et al., 2019; Signorelli et al., 2019). SKU5 may impact this mechanism indirectly through affecting the action of ABP1, and a reduction of the auxin signaling into TOR would shift the balance in favor of stronger ABA responses, such as that seen in the spaceflight acclimation of *sku5*. A ROP6 pathway also mediated by ABP1 affects the activity of KATANIN, which severs cortical microtubules to facilitate reorganization of the microtubule network in response to environmental stimuli (Chen et al., 2016). As a result, the *spr1* mutant may be faster to form a spaceflight-tolerant microtubule network due to its altered microtubule kinetics, while the *sku5* mutant may not be able to perform this function as rapidly due to altered ABP1 signaling.

#### *Spr1* Mutation Produces a Decrease in Differential Gene Expression in Spaceflight

The spaceflight DEGs of *spr1* and Col-0 were primarily in the defense and salicylic acid-mediated signaling related to cell wall remodeling (Figures 5B, 6B). Genes related to these cell wall pathways are common in spaceflight transcriptomes (Paul et al., 2012b, 2013, 2017; Correll et al., 2013; Kwon et al., 2015; Johnson et al., 2017). While plants grown in microgravity may be more susceptible to pathogenic infection (Leach et al., 2001; Ryba-White et al., 2001), the fact that all examples to date of upregulated pathogen response genes are from plants grown in sterile conditions suggests that these genes are serving other purposes of cell wall remodeling in spaceflight (Paul et al., 2012b, 2013, 2017; Correll et al., 2013; Choi et al., 2019). ALD1 is a positive regulator of salicylic acid accumulation pathways through the generation of metabolites (Nie et al., 2011; Cecchini et al., 2015). NIMIN1 and DLO1 contribute to opposing processes, acting to repress immune responses at the level of expression regulation (Kohler et al., 2002; Zhang et al., 2013; Zeilmaker et al., 2015). Salicylic acid induces the lipid raft-localized pathways induced by ABA, in order to promote cellular uptake of water (Demir et al., 2013; Prodhan et al., 2018). The upregulation of salicylic acid signaling in *spr1* may act through these pathways, promoting abiotic stress response through pathways normally associated with defense responses (Figures 5B, 6B). This hypothesis might also explain why the induction of “defense” and wound response pathways occurs in spaceflight in the absence of pathogenic infections (Nejat and Mantri, 2017). The recently characterized ORBITALLY MANIFESTED GENE 1, a regulator of ROS signaling that is both flg22- and wound-inducible, provides a spaceflight-relevant example (Sng et al., 2018). Considering that wounding and pathogen invasion also mechanically disrupt membranes and cell walls (Chen et al., 2016), these pathways may be signaling remodeling of cellular structures and the microtubule network in spaceflight. Flg22 application to Arabidopsis cell cultures and induction of MAMP-triggered immunity also has the effect of redistributing flavonoid compounds from biosynthetic processes important to oxidative stress response processes to the production of compounds important to defense (Schenke et al., 2019). Genes associated with the production of phenylpropanoids are less-represented among the *spr1* DEGs downregulated in spaceflight compared to Col-0, although they are also more highly represented among *spr1* DEGs downregulated across development in spaceflight (Figures 6B, 7B). As such, these DEGs negatively regulating defense signaling in *spr1* may contribute to an enhanced ability to mediate ROS generated in spaceflight through the redistribution of limited resources early in its development. This conclusion is also supported by the gene categories enriched in the spaceflight acclimations of Col-0 and *spr1*, where DEGs associated with responses to oxidative stress are more enriched among genes upregulated in FLT in *spr1* than in Col-0 (Figure 6B).

Many abiotic stresses trigger remodeling of cortical microtubules, with salt stress tolerance requiring the 26S proteasome-mediated degradation of SPR1 (Shoji et al., 2006; Wang et al., 2011; Chen et al., 2016). Microtubule reorientation also contributes to cell elongation in the spaceflight response of hypocotyls (Soga et al., 2018). The *spr1* mutant, which has altered microtubule dynamics in favor of decreased stability, may have enhanced capabilities for remodeling of microtubule networks in the response to many forms of environmental stress (Galva et al., 2014).

#### *Spr1* and *Sku5* Mutants Both Differentially Regulate Skewing-Associated Genes in Response to Spaceflight

Despite the many differing spaceflight responses of *spr1* and *sku5*, DEGs identified as likely to be associated with the integrative signaling of skewing (Schultz et al., 2017) were among those differentially expressed in both mutants in spaceflight (Figure 13). Two of the highly likely skewing genes, SENESCENCE 1 (SEN1) and ASPARAGINE SYNTHETASE 1 (ASN1) are induced in spaceflight (Figure 13). The SKU5 protein is endocytosed through different pathways within the root tip dependent on local auxin signals and perceived stress conditions (Baral et al., 2015). SKU5 could act to modulate growth regulation through its aforementioned association with the ABP1 apoplastic signaling pathway upstream of TOR, and both SKU5 and SPR1 can affect microtubule reorganizing pathways. This would enable SKU5 to impact the spaceflight response via the regulation of meristematic competence and cell cycle progression, processes that are affected in spaceflight (Matía et al., 2010), clinorotation, RPM exposure, and magnetic levitation (Herranz and Medina, 2014; Herranz et al., 2014; Boucheron-Dubuisson et al., 2016). This is supported by the M-phase peak of SKU5 expression and its previously hypothesized role in regulating the cell cycle (Menges et al., 2002; Sedbrook et al., 2002). However, the importance of senescence in the context of root skewing has not been well-studied. The recent association of strigolactone-independent karrikin-sensing pathways with skewing provides a potential direction for study (Swarbreck et al., 2019). The loss of the protein MORE AXILLARY BRANCHES 2 (MAX2), which also participates in strigolactone-associated senescence signaling, exacerbates the skewing phenotype of *sku5* in a *sku5 max2* line (Ueda and Kusaba, 2015; Swarbreck et al., 2019). Therefore, these results reinforce connections between environmental and senescence signaling and root skewing. The association of skewing-associated genes with these regulatory networks provide a means by which the mutation of these genes can alter the response to the spaceflight condition.

## Conclusion

The physiological adaptation of *sku5* to spaceflight is characterized by powerful stress responses, as well as the strong induction of LEAs and other genes associated with seed development. In contrast to *sku5*, *spr1* differentially expressed fewer genes than Col-0 to physiologically adapt to spaceflight. This observation suggests that the *spr1* mutation imbued an enhanced ability for rapid spaceflight acclimation while *sku5* required an extended spaceflight response to accomplish acclimation, even to the point of initiating classic deep stress responses. These observations further suggest that the functional distinctions between *sku5* and *spr1* inform distinct aspects of the spaceflight response in plants, in particular the responses of young seedlings. The dramatic responses of *sku5* suggest that lipid raft nanodomains of the plasma membrane and their associated GPI-APs play important roles in spaceflight physiological adaptation.

The fact that *sku5* and *spr1* show markedly different gene expression patterns in spaceflight suggests that the two skewing pathways highlighted by SKU5 and SPR1 differentially affect the mechanisms used by plants to physiologically acclimate to spaceflight. Since *spr1* and *sku5* root lengths are not altered by spaceflight, the effects of these mutations lie not in the process of growth, but in the processes that direct morphology and structure, likely tying together the large number of cell wall remodeling genes observed as differentially expressed in spaceflight. The data from APEX-03-2 therefore provide an initial functional dissection of the spaceflight response in roots, one that allows an integration of pathways associated with root directionality, morphology and physiological responses to spaceflight.

## Data Availability Statement

The datasets generated and analyzed, composed of the RNA-Seq data and full output of the differential gene expression pipeline, for this study can be found in the Gene Expression Omnibus (GEO – https://www.ncbi.nlm.nih.gov/geo/) under the accessions GSE95620 (https://www.ncbi.nlm.nih.gov/geo/query/acc.cgi?&acc=GSE95620) and GSE95582 (https://www.ncbi.nlm.nih.gov/geo/query/acc.cgi?acc=GSE95582), and also in NASA’s GeneLab Database (GLDS-218). A presentation outlining the experimental process and which contains video data of the harvests can be found at the NASA LSDA (Life Sciences Data Archive (https://lsda.jsc.nasa.gov/Dataset/dataset_info/13840).

## Author Contributions

A-LP and RF conceived the experiments and acquired funding for the experiments. A-LP, RF, NS, and AZ prepared and carried out the experiments. All authors contributed to the data analyses. BC, A-LP, and RF prepared the manuscript.

## Conflict of Interest

The authors declare that the research was conducted in the absence of any commercial or financial relationships that could be construed as a potential conflict of interest.

## Abbreviations

d: day
DEG: differentially expressed gene
FLT: spaceflight
GC: ground control
GO: gene ontology
GPI-AP: glycosylphosphatidylinositol-anchored protein.
