# Supplementary material for: Root Skewing-Associated Genes Impact the Spaceflight Response of Arabidopsis thaliana
Source: Front Plant Sci. 2020 Mar 4;11:239. doi: 10.3389/fpls.2020.00239 (PMC7064724; doi:10.3389/fpls.2020.00239)
Supplement: Supplementary file 1 [file Data_Sheet_1.zip › Table S1.DOCX]

**Table S1.** The elapsed time between plate opening and completion of KFT actuation in spaceflight. The elapsed time indicated encompasses the time between the opening of each plate for imaging and harvest, and the completion of KFT actuation. The elapsed time was note recorded for the 8d WS harvests. The Ground Controls were harvested in comparable windows of time, but the precise elapsed time was not recorded.

| **Genotype** | **4 day old** | **8 day old** |
| --- | --- | --- |
| Col-0 | 3 min., 34 sec. | 2 min., 42 sec. |
| *spr1* | 2 min., 52 sec. | 2 min., 31 sec. |
| WS | 3 min., 21 sec. | Data Unavailable |
| *sku5* | 2 min, 32 sec. | 2 min., 45 sec. |
