# Supplementary material for: Root Skewing-Associated Genes Impact the Spaceflight Response of Arabidopsis thaliana
Source: Front Plant Sci. 2020 Mar 4;11:239. doi: 10.3389/fpls.2020.00239 (PMC7064724; doi:10.3389/fpls.2020.00239)
Supplement: Supplementary file 1 [file Data_Sheet_1.zip › Table S2.DOCX]

| Genotype | Germination Rate - GC | *n*, GC | Germination Rate - FLT | *n*, FLT | χ^2^, GC | χ^2^, FLT |
| --- | --- | --- | --- | --- | --- | --- |
| Col-0 | 100% | 51 | 100% | 42 |  |  |
| *spr1* | 100% | 44 | 100% | 36 |  |  |
| WS | 93.48% | 46 | 85.42% | 48 |  |  |
| *sku5* | 76.60% | 47 | 47.73% | 44 |  | * |

Supplementary Table 2: Germination rates observed from APEX-03-2 4-day harvest images.

Abbreviations: GC – Ground Control, FLT – Spaceflight, WT – Wild Type; * indicates *p* < 0.01
